# Evaluation of Leaching Characteristics of Heavy Metal Ions from Red Mud–Graphite Tailings

**DOI:** 10.3390/toxics13030211

**Published:** 2025-03-14

**Authors:** Kangli Li, Xiaolei Lu, Congcong Jiang, Dan Wang, Jiang Zhu, Meiling Xu, Lina Zhang, Xin Cheng

**Affiliations:** 1 Shandong Provincial Key Laboratory of Green and Intelligent Building Materials, University of Jinan, Jinan 250022, China; lkl5552022@163.com (K.L.); mse_luxl@ujn.edu.cn (X.L.); mse_jiangcc@ujn.edu.cn (C.J.); mse_wangd@ujn.edu.cn (D.W.); mse_zhuj@ujn.edu.cn (J.Z.); chm_xuml@ujn.edu.cn (M.X.); 2 School of Materials Science and Engineering, University of Jinan, Jinan 250022, China

**Keywords:** red mud–graphite tailings, heavy metal stabilization, semi-dynamic leaching

## Abstract

The rapid growth of aluminum and graphite industries has generated substantial stockpiles of red mud and graphite tailings, which pose environmental risks due to their high heavy metal content and potential for soil and water contamination. This study investigated the leaching behavior of heavy metals from these materials post-stabilization using cement and a sulfonated oil-based ion curing agent, thereby evaluating their suitability for safe reuse. Semi-dynamic leaching experiments were employed to measure heavy metal release, supplemented by kinetic modeling to discern key leaching mechanisms. The findings indicated that the heavy metal concentrations in leachates were consistently below regulatory standards, with leaching dynamics influenced by dual mechanisms: the diffusion of ions and surface chemical reactions. A diffusion coefficient-based analysis further suggested low leachability indices for all metals, confirming effective immobilization. These results suggest that cement and curing agent-stabilized red mud–graphite tailing composites reduce environmental risks and possess characteristics favorable for resource recovery, thus supporting their sustainable use in industrial applications.

## 1. Introduction

An escalation in the exploitation and utilization of mineral resources has emerged with the rapid advancement of industry, concurrently presenting a spectrum of issues. In 2018, the United Nations Environment Programme and the International Solid Waste Association co-authored the report titled “What a Waste 2.0: A Global Snapshot of Solid Waste Management to 2050”, which highlights the substantial global generation of industrial solid waste at approximately 48.7 kg per capita per day and underscores the urgent challenges of addressing solid waste management [1]. Improper disposal of industrial solid wastes can lead to severe environmental contamination. At present, common industrial solid wastes encompass red mud, graphite tailings, iron tailings, flotation waste, gangue, metallurgical slag, and so forth [2,3]. Among these, red mud and graphite tailings have drawn great attention due to their tough utilization treatment. Red mud, also called bauxite residue, is a byproduct generated from bauxite with strong alkalinity during alumina extraction [4]. It is characterized by its large yield, as well as enrichment in diverse heavy metal ions, including chromium (Cr), cadmium (Cd), lead (Pb), and mercury (Hg) [5,6]. Failure to appropriately manage red mud can result in the leaching of these heavy metal ions into the environment, leading to significant pollution of soil, water bodies, and ecosystems. As a significant resource, graphite is extensively used in fields such as new energy, electronics, and aerospace [7]. However, only 10–15% of graphite purified by flotation can be used, resulting in the generation of graphite tailings [8]. Comprehensive utilization of red mud and graphite tailings not only mitigates waste accumulation but also decreases environmental pollution risk. However, the remediation of heavy metal ions in these tailings is crucial for their safe utilization.

Solidification/stabilization (S/S) technology is a mature remediation method for addressing heavy metal pollution [9,10,11]. Based on the type of curing/stabilizing agent employed, heavy metal treatments can be commonly categorized into cement S/S [12,13], sulfide S/S [14,15], and biological S/S [16,17]. Among them, cement S/S is most prevalent due to its cost-effectiveness and ease of use. This technique involves mixing heavy metal contaminants with cement, utilizing the alkaline environment and hydration products (i.e., C-S-H gel and ettringite) from the hydration reaction of cement to chemically bind and physically encapsulate the heavy metals, thereby diminishing their environmental mobility and bioavailability [18,19,20]. Nevertheless, the cement-solidified material may experience environmental stresses, including acid, alkali, and salt attack, and over extended use, which can diminish the durability of cement and the long-term stability of bound heavy metals [21,22]. Consequently, a search is required for a product capable of enhancing the stability of cement-based S/S of heavy metal ions.

Addressing this demand, Ma et al. demonstrated an innovative approach in which organic ion-curing agents were integrated with cement to treat organically contaminated soils, achieving concurrent immobilization of both heavy metals and organic pollutants through ionic chelation and pore structure refinement [23]. This approach demonstrates a potential way of improving the stability of cement-based S/S for heavy metal ions. The use of an organic ion curing agent in conjunction with cement not only helps to reduce ion exchange but also enhances the overall performance of the solidified material. At the same time, ionic soil stabilization (ISS) as an effective curing agent has attracted extensive research for its ability to remediate heavy metal ions in soil [24,25,26]. Its mechanism primarily involves reducing soil particle surface charge via ion exchange, thinning the double-layer electrical potential, and increasing particle intergranular forces, thereby improving the compaction and strength of soil. However, further research is needed to explore the full potential of this method and to optimize the formulation of the organic ion curing agent for different types of heavy metal ions. In light of the efficacy of the combined ISS–cement curing technology for treating contaminated soil [27,28,29], our research group has further refined the design and independently formulated a sulfonated oil-based ISS. This formulation comprises sulfonated oil and specific quantities of Ca^2+^, Mg^2+^, and Na^+^ ions, details of which have been detailed in our prior publication [30].

The characteristics of fine particles and high moisture content of red mud make it relatively sticky [31]. Graphite tailings, resulting from the silica residue of graphite beneficiation, are coarse particles with a loose structure and are deficient in gelling activity [32]. Whilst they are difficult to use separately, the coarse particles of graphite tailings can be potentially embedded into the fine particles of red mud as skeletons to acquire good gradation and mechanical properties of the finished structure. Therefore, this study delved into the potential of ISS combined with cement in the remediation of a mixed system of red mud and graphite tailings. The effects of ISS and diverse environmental conditions on the leaching of harmful metals such as Cr, Pb, and Cu from red mud–graphite tailings were studied. Additionally, the post-curing leaching parameters were compared and assessed using models such as the unreacted shrinkage core, Elovich, double constant, and Avrami models, to identify the most appropriate model for describing the leaching behavior of heavy metal ions in red mud–graphite tailings. Moreover, the leachability index(LX) for each metal was calculated utilizing the diffusion coefficients to assess the sustainability of reusing the red slime–graphite tailings following S/S processes.

## 2. Materials and Methods

### 2.1. Materials

Red mud was obtained from Shandong Weiqiao Pioneering Group Co., Ltd., (Binzhou, China). Graphite tailings were collected from Pingdu graphite plant, (Qingdao, China). Ordinary Portland cement 42.5 (equivalent to European CEM I 42.5) was acquired from Jining Conch Cement Group Co., Ltd., (Jining, China). The ionic soil stabilizer, sulfonated oil, as the main content, was synthesized in the laboratory via the reaction of palmitic acid and sulfuric acid; the relevant methods of preparation and the properties of the stabilizer are outlined in a previous research report [30].

### 2.2. Specimen Preparation

The preparation of the test specimen followed the procedures detailed in the standard JTG E51-2009 [33]. Graphite tailings and red mud were dried in a dryer at 50 °C for 24 h; those with a particle size exceeding 4.75 mm were screened out. First, 18 portions of red mud and graphite tailings at a weight ratio of 1 were mixed thoroughly with 2.4 portions of water. One portion of cement was then added one hour before forming the test piece and mixed uniformly to ensure homogeneity. The compacted mixture was cast into a cylindrical mold (Φ × H = 50 × 50 mm). Specimens were removed from the mold with a hydraulic jack, weighed, sealed in plastic bags, and transferred to the curing room (temperature: 20 ± 2 °C; relative humidity: >95%).

### 2.3. Test Methods

#### 2.3.1. Chemical Composition

The elemental and mineral compositions of various samples were assessed using X-ray fluorescence (XRF) (BRUKE, Karlsruhe, Germany) and X-ray diffraction (XRD) (BRUKER, Karlsruhe, Germany) techniques. The XRD analysis was performed using a diffractometer equipped with CuKα radiation, at a voltage of 40 kV and a current of 20 mA within a 2θ scan range from 5° to 80°. The sample was dried to a constant weight at 105 °C using a dryer and subsequently cooled to room temperature. It was then crushed into powder in an agate mortar. The resulting powder was sieved through a mesh with a size of 0.075 mm. To mitigate sample contamination, the powders were stored in hermetically sealed barrier bags prior to conducting chemical composition analysis.

#### 2.3.2. Microstructure Characterization

To ascertain the microstructural variations in red mud and graphite tailings samples post-cement and ISS curing, the samples were analyzed using scanning electron microscopy (SEM) (ZEISS, Jena, Germany). The preparation procedure for SEM samples involved the following steps: (1) drying the samples at 105 °C for 6 h in a dryer; (2) attaching each sample to the sample stage with conductive tape; and (3) applying a thin layer of platinum to the sample surface via sputtering, thereby mitigating the charge effect.

#### 2.3.3. Heavy Metal Leaching Tests

Semi-dynamic leaching tests were conducted following the US EPA 1315 method [34]. Polypropylene buckets with 1 L capacities were selected as extraction vessels to facilitate adequate contact between the extraction liquid and the sample, thus ensuring effective leaching. The liquid–solid ratio between the extraction liquid and the sample surface was maintained at 8.45 mL/cm^2^. The leaching periods were set at 1 day (d), 3 d, 7 d, 10 d, 14 d, 21 d, 28 d, and 35 d. Following each period, the leachate was filtered using a 0.45 μm pore-size filter membrane to eliminate any suspended particles that could compromise the analytical results. The filtered leachate was subsequently acidified to facilitate further analysis. An inductively coupled plasma optical emission spectrometer (ICP-OES, ICAP7200) (Thermo Fisher, Waltham, MA, USA) was utilized to quantify the concentration of heavy metals in the leachate, with a detection limit of 0.01 mg/L, ensuring precise elemental concentration measurements. The average of three replicate samples was employed to guarantee the representativeness and reliability of the data. In the semi-dynamic leaching tests, a leachate with a pH of 3.2 (±0.05) was prepared by diluting a mixed solution of sulfuric acid and concentrated nitric acid in a 2:1 molar ratio, given that acid rain in China is predominantly due to acidic gases, including sulfur dioxide (SO_2_) and nitrogen oxides (NO_X_) [35]. The control group for the other leachate was prepared using deionized water.

### 2.4. Data Analysis

The cumulative leaching mass of the solute per unit area was calculated using Equation (1):

(1) $$M_{t}=2\rho C_{0}{\left(\frac{D_{\mathrm{obs}}t}{\pi }\right)}^{1/2}$$

where M_t_—cumulative mass released during per unit leaching interval (mg/m^2^); ρ—density of the sample (kg/m^3^); C_0_—initial solute concentration of the solid matrix (mg/kg); D_obs_—observed diffusivity of solute in leachate (m^2^/s); t—leaching time (s). To facilitate analysis, the logarithm of Equation (1) was computed as follows:

(2) $$\mathrm{lg}M_{t}=\mathrm{lg}\left[2\rho C_{0}{\left(\frac{D_{\mathrm{obs}}t}{\pi }\right)}^{1/2}\right]+0.5\mathrm{lgt}$$

The interval mass released can be calculated for each leaching interval as follows:

(3) $$M_{\mathrm{ti}}=\frac{\;C_{i}V_{i}}{A}$$

where M_ti_—mass released during the current leaching interval (mg/m^2^); C_i_—constituent concentration in the eluate for the interval (mg/L); V_i_—eluate volume in the interval (L); A—external geometric surface area of the specimen exposed to the eluent (m^2^).

An observed diffusivity can be determined using the logarithm of the cumulative release plotted against the logarithm of time. When the slope of lgM_t_ − lgt is 0.35–0.65, the mechanism is diffusion; when the slope is <0.35, the mechanism is surface wash-off; and when the slope is >0.65, the mechanism is dissolution [36]. An observed diffusivity can then be determined for each leaching interval using Equation (4):

(4) $$D_{i}^{\mathrm{obs}}=\pi {\left[\frac{M_{\mathrm{ti}}}{2\rho C_{0}(\sqrt{t_{i}\;}-\sqrt{t_{i-1}})}\right]}^{2}$$

where $D_{i}^{\mathrm{obs}}$—observed diffusivity for the leaching interval (m^2^/s); t_i_—cumulative contact time at the end of the current leaching interval (s); t_i−1_—cumulative contact time at the end of the previous leaching interval (s).

Based on Equation (4), the D_obs_ were calculated as follows:

(5) $$D_{\mathrm{obs}}=\frac{\;\sum_{i}^{n}D_{i}^{\mathrm{obs}}}{n}$$

The leachability index was calculated based on $D_{i}^{\mathrm{obs}}$ values:

(6) $$\mathrm{LX}=\frac{1}{n}\sum_{i=1}^{n}\left(-\mathrm{lg}D_{i}^{\mathrm{obs}}\right)$$

## 3. Leaching Models

In this section, mathematical conceptual models that represent various leaching mechanisms are formulated to estimate the leaching parameters and kinetics for different heavy metals as a function of time.

### 3.1. Unreacted Shrinking Core Model (USCM)

The USCM is widely used to describe the behavior of solid particles during chemical reactions. The leaching process of a liquid–solid reaction can be represented by the USCM [37]. If the reaction is controlled by internal diffusion, the rate equation is as follows [38,39]:

(7) $$1-\;2/3X-{(1-X)}^{2/3}=k_{r}t$$

If the reaction is controlled by interfacial chemistry, the rate equation is as follows:

(8) $$1-{(1-\;X)}^{1/3}=k_{d}t$$

If the reaction is controlled by a mixture of interfacial chemistry and diffusion, the rate equation is as follows:

(9) $$1/3\ln(1\;-\;X)+{(1-X)}^{1/3}-1=k_{m}t$$

In Equations (7)–(9), X is the percentage of leaching; k_r_, k_d_, and k_m_ are the rate constants calculated from Equations (7)–(9), respectively; and t is the leaching time.

### 3.2. Double-Exponential Model

The essence of the double exponential equation is the modified Freundlich equation, which is an empirical expression. The study shows that the Freundlich dynamic equation can be used to infer the adsorption and desorption kinetics of anion ions and heavy metals in soil and to deduce the correlation coefficient of the adsorption process [40]. It is suitable for more complex kinetic processes. The expression is as follows:

(10) lnC=a+blnt

In Equation (10), C is the concentration of the heavy metal in the solution at time t; a and b are the fitting constants; and t is the leaching time.

### 3.3. Elovich Equation

The Elovich equation is frequently employed to characterize the progression of a series of reaction mechanisms, including the diffusion of solutes within the solution phase or at the interface, and the activation and deactivation of surface processes [41,42,43]. The results show that the Elovich equation can reflect chemisorption and desorption processes, especially heterogeneous chemical reactions, and can describe the irregularity neglected by other kinetic equations.

The expressions of the equation are as follows:

(11) $$C_{t}=a_{1}+b_{1}\mathrm{lnt}$$

where C_t_—cumulative leaching amount of heavy metals at time t; a_1_ and b_1_—constants, t—leaching time. Constant b_1_ represents the rate of diffusion of heavy metals from the solid phase to the liquid phase. A higher value of b_1_ indicates a faster diffusion rate [44,45].

### 3.4. Avrami Model

The Avrami equation describes isothermal solid-state transformation reactions based on nucleation and growth kinetics. This equation can describe the change in the volume fraction of solid materials with time during phase transition, consistent with the solid particle surface gradually dissolving into the solution in the leaching reaction. Therefore, the Avrami equation (Equation (12)) can describe the reaction kinetics of leaching. It is successfully applied to calculate for leaching kinetics of mixed metal oxides [46,47].

The Avrami kinetic model is given in Equation (12).

(12) $$-\ln\left(1\;-\;X\right)=kt^{n}$$

where: n—Avrami exponent, and k is the constant.

## 4. Results and Discussion

### 4.1. Elemental Composition

The elemental compositions of red mud and graphite tailings are listed in Table 1. In red mud, iron (Fe) is the most abundant element, making up 36.6% of the total mass, followed by aluminum (Al) at 22.91% and silicon (Si) at 11.62%. These three elements alone account for over 71.13% of the total mass of red mud. On the other hand, graphite tailings are dominated by Si, which constitutes 39.37% of the total mass, with Fe at 11.55% and Al at 11.87%, together making up 62.79% of the total mass.

### 4.2. Mineral Composition

**Figure 1:**
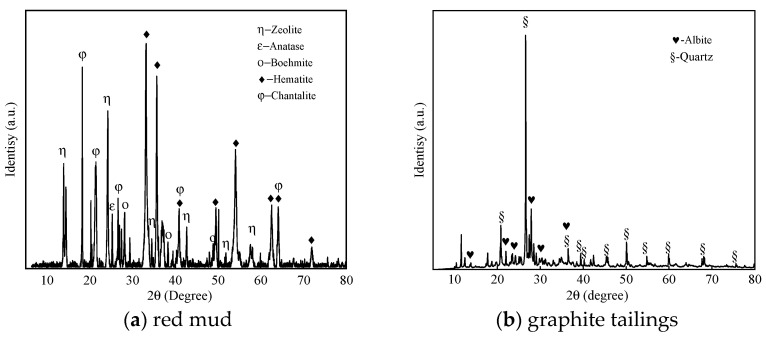
XRD pattern of red mud and graphite tailings: (**a**) red mud, (**b**) graphite tailings.

Figure 1 illustrates the XRD patterns obtained from red mud and graphite tailings. Red mud primarily consists of hematite, zeolite, and boehmite, consistent with previous reports [48]. Graphite tailings predominantly contain quartz and albite, a composition documented in the literature [49].

### 4.3. Microstructure Characteristics

**Figure 2:**
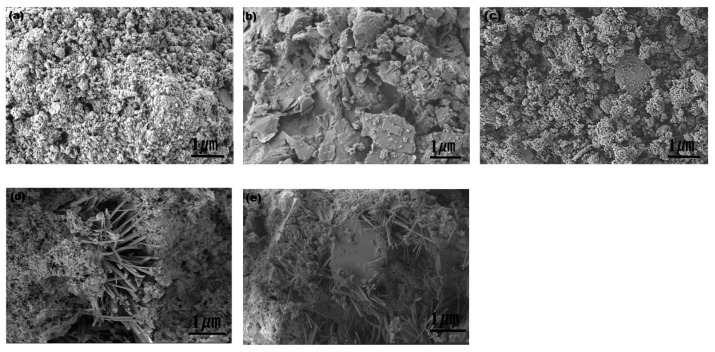
SEM images of red mud and graphite tailings: (**a**) red mud, (**b**) graphite tailings, (**c**) red mud and graphite tailing, (**d**) red mud graphite tailings with cement, (**e**) red mud graphite tailings with cement and ISS.

Figure 2 illustrates the microscopic diagrams of red mud, graphite tailings, and the mixture of red mud and graphite tailings with cement and ISS. Figure 2a,b depict the disparities in particle size and morphology between red mud and graphite tailings, revealing that red mud exhibits smaller particle sizes predominantly in a granular form, whereas graphite tailings are predominantly in flake form. These observations correlate with the outcomes of the XRD analysis. Figure 2c shows that in the absence of cement, the red mud and graphite tailings underwent only physical mixing, with no notable chemical interaction. Conversely, the inclusion of cement (Figure 2d) triggered a hydration reaction, yielding hydration products that facilitated direct bonding of the materials, thereby significantly enhancing their bonding attributes. Ultimately, Figure 2e demonstrates that the addition of ISS further stimulates the generation of cement hydration products. This is attributed to ISS’s ability to improve the hydration environment and provide additional active sites, thereby accelerating the hydration reaction [50]. This discovery underscores the potential of ISS to enhance the properties of cement-based materials, particularly in improving bonding characteristics.

### 4.4. Leaching Characteristics

The leaching of selected heavy metals, such as Cr, Pb, and Cu, from red mud–graphite tailings was investigated using a simulated environment experiment.

#### 4.4.1. Leachability of Cr, Pb, and Cu

**Figure 3:**
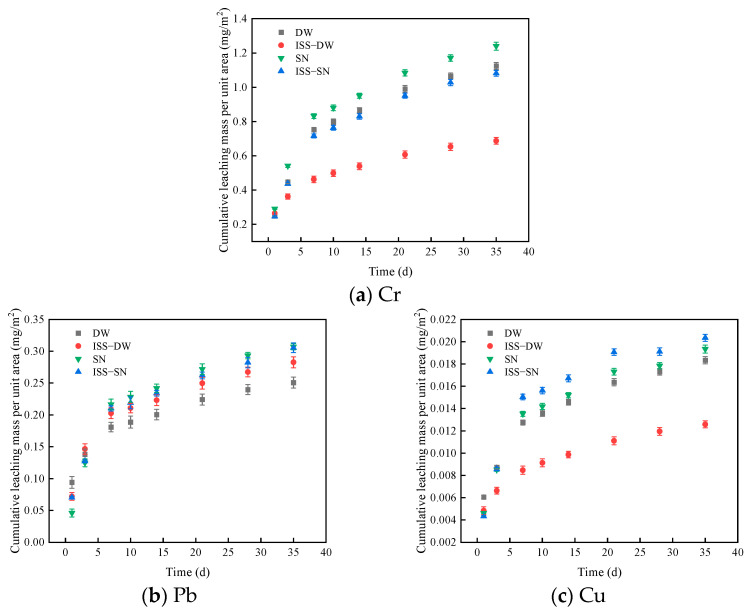
The cumulative leaching mass of (**a**) Cr, (**b**) Pb, and (**c**) Cu. DW represents deionized water as the leaching solution of the test block; ISS-DW refers to the reagent ISS added during the preparation of the test block, and the leaching solution is deionized water; SN indicates that the leaching solution of the test block is the mixed solution of sulfuric acid and nitric acid; and ISS-SN indicates that the reagent ISS is added during the preparation of the test block, and the leaching solution is the mixed solution of sulfuric acid and nitric acid.

To evaluate the potential environmental risks associated with the use of red mu–graphite tailings, we initially compared leaching data in Figure 3 against a related standard (GB5085.3-2007, ‘Leaching Toxicity Identification of Hazardous Waste [51]’). The study revealed that the concentrations of heavy metals in all samples were below the hazardous waste limits where the concentration limits of Cr, Pb, and Cu are referenced to be 15 mg/kg, 5 mg/kg, and 100 mg/kg, respectively.

Figure 3 illustrates the cumulative leaching amounts of Cr, Pb, and Cu. The highest leaching concentration was observed for Cr followed by Pb, and the lowest was for Cu. The leaching curve revealed an increase in heavy metal leaching with time, particularly during the initial stages, where the curve exhibited a sharp upward trend before leveling off. The initial increase in leaching concentrations was attributed to the structural cracking that occurred in the test samples as they absorbed water. This cracking expanded the contact area between the samples and the leaching solution, which in turn promoted the dissolution of Cr, Pb, and Cu ions [52].

Furthermore, heavy metal leaching concentrations were elevated in acidic environments compared to deionized water environments, indicating that acidic conditions promoted heavy metal leaching, thus amplifying environmental risks [53,54].

The incorporation of ISS reduced heavy metal leaching concentrations, indicating that ISS effectively suppressed the leaching of Cr, Pb, and Cu ions, thereby mitigating environmental risks. Notably, in the deionized water environment, Pb exhibited different behavior, with the sample without ISS showing a lower leaching concentration. This discrepancy arose from ISS altering the chemical compound of Pb, which facilitated its dissolution [55].

In conclusion, while the leaching of heavy metals from red mud and graphite tailings increased over time, concentrations remained below the hazardous waste standard, indicating manageable environmental risks. The addition of ISS was effective in reducing heavy metal leaching; however, further investigation is required to elucidate the mechanism of its impact on Pb in the deionized water environment.

#### 4.4.2. Leaching Kinetics and Mechanisms

**Figure 4:**
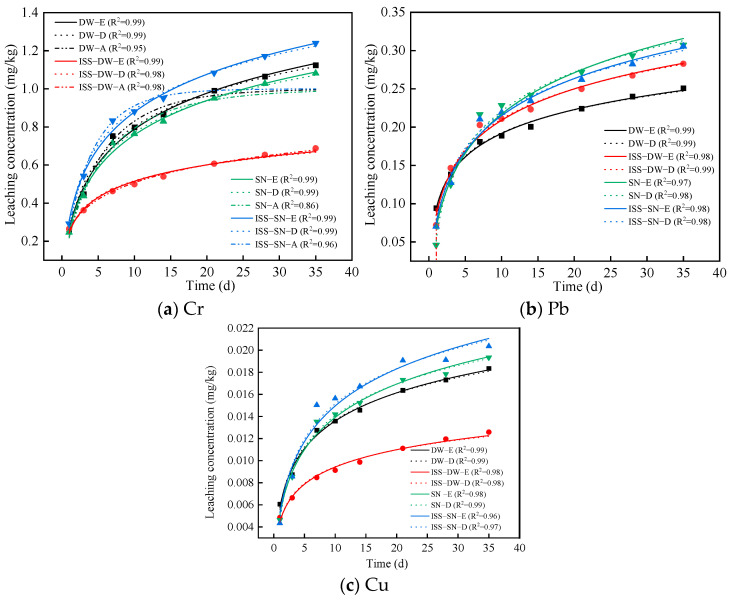
Model fitting diagram of Cr, Pb, and Cu form leaching experiment: (**a**) Cr, (**b**) Pb, (**c**) Cu.

In the assessment of environmental risks associated with red mud–graphite tailings, the leaching model outlined in Section 2 was utilized to thoroughly describe the leaching process. The fitting results and parameters of the USCM offered insights into the leaching behavior of heavy metal ions in various environmental conditions. Table 2 details the fitting parameters for these models while Figure 4 illustrates the fitting outcomes for Cr, Pb, and Cu ions under the Elovich, double constant, and Avrami models.

Figure 4 and Table 2 reveal that the leaching of Cr ions in red mud graphite tailings is primarily controlled by internal diffusion. This indicates that in the absence of ISS, the release of Cr ions is limited by their migration rate within the solidified matrix. For Cr ions in the deionized water environment, the leaching was influenced by a combination of surface chemical reactions and diffusion. The addition of ISS altered the leaching mechanism, resulting in a process where Cr ions were affected by both internal diffusion and the rate of surface chemical reactions. The leaching of Cr ions was found to be in accordance with the Elovich, double exponential, and Avrami equations, which are typically used to characterize solid–liquid interface reactions and diffusion-controlled processes.

The leaching of Pb and Cu ions was also dominated by a mix of surface chemical reactions and diffusion, similar to the behavior observed for Cr ions after adding ISS addition. This combined control is likely associated with ISS, which could have enhanced surface chemical reactions while modifying the diffusion pathway of ions. The leaching of Pb and Cu ions was consistent with the Elovich and double exponential equations, further emphasizing the importance of surface chemical reactions and diffusion in their leaching.

**Figure 5:**
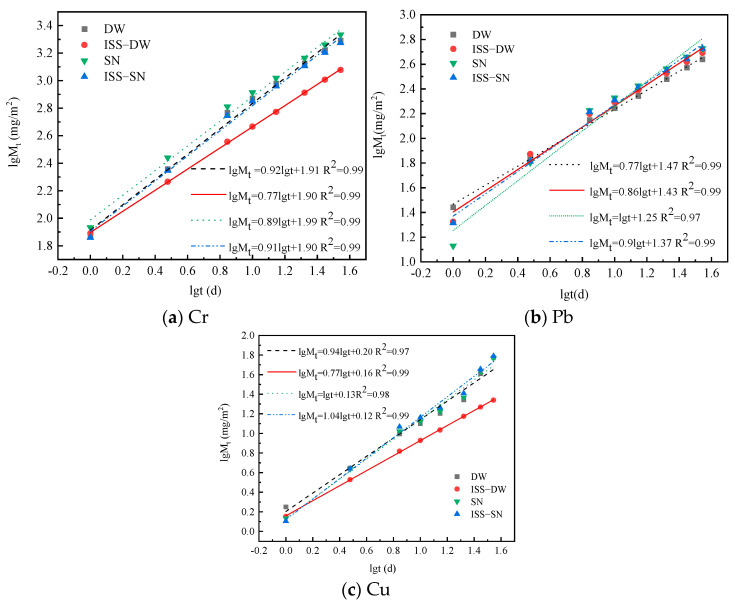
Leaching mechanisms of (**a**) Cr, (**b**) Pb, and (**c**) Cu.

The relationship between cumulative leaching mass and cumulative leaching time for Cr, Pb, and Cu under varying conditions is delineated in the double logarithmic coordinate system depicted in Figure 5. Analysis of these graphs elucidates the leaching kinetics within the test samples, providing a comprehensive understanding of the leaching dynamics for Cr, Pb, and Cu. The slopes depicted in Figure 5, with values exceeding 0.65, indicate that the leaching of these heavy metals is not purely diffusive but also encompasses a dissolution component [56]. This dissolution component was likely influenced by the chemical composition of the test sample, its pore structure, and the interactions between the heavy metal ions and the curing agent.

The inclusion of a dissolution process in the leaching mechanism suggests that heavy metal ions can more readily be released from the solid matrix at the onset of leaching. This release is likely facilitated by the rapid desorption of surface-bound ions or the quickened pace of surface chemical reactions, which hastens the transition of these ions from the solid phase into the leaching solution. Understanding these nuances in the leaching process is pivotal for the development of effective ISS and environmental management strategies for mitigating potential environmental risks associated with the disposal or utilization of red mud–graphite tailings.

**Figure 6:**
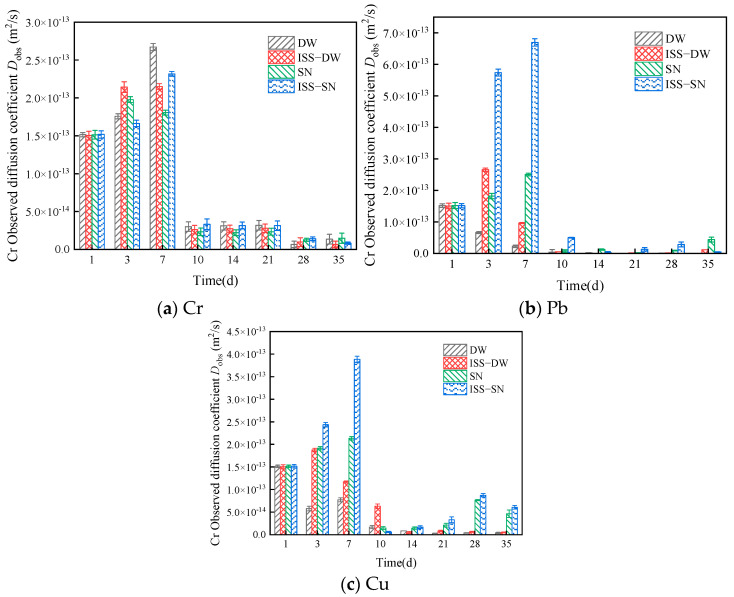
Observed diffusion coefficients of (**a**) Cr, (**b**) Pb, and (**c**) Cu.

The D_obs_ were calculated using Equation (5) and are depicted in Figure 6. The leaching value for Cr ions in the initial 7 days was an order of magnitude greater than that in the subsequent period (10^−14^–10^−13^), and the D_obs_ tends to stabilize over time. A similar trend was observed for the leaching of Pb and Cu. The results indicated a faster initial leaching rate for these ions, validating the initial observations. As indicated in Figure 6, the D_obs_ for Cr varied from 6.69 × 10^−15^ to 1.51 × 10^−13^ m^2^/s, which is smaller than the D_obs_ values reported by Zhang et al. [57]. The D_obs_ values for Cu and Pb were also lower than those reported by Shi et al. and Sun et al., respectively [58,59].

**Figure 7:**
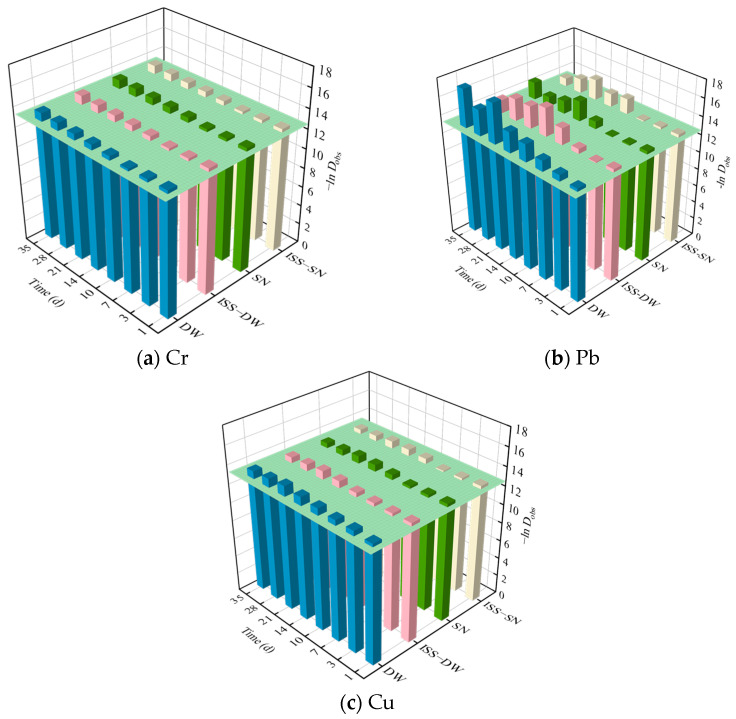
−lnDobs of (**a**) Cr, (**b**) Pb, and (**c**) Cu.

These results confirmed the effective inhibition of heavy metal migration through stabilization/solidification (S/S). Furthermore, the data demonstrated that the inhibitory effects of different curing materials on heavy metals vary significantly. As Malviya and Chaudhary proposed [60], the mobility of heavy metals was quantified through their leaching rate, represented by the negative natural logarithm of the observed dissolution rate (−ln D_obs_). A value of −ln D_obs_ > 12.5 indicated low mobility, 11.0 < −ln D_obs_ < 12.5 suggested moderate mobility, and −ln D_obs_ < 11.0 denoted high mobility. The leaching rates of Cr, Pb, and Cu shown in Figure 7 were determined using Equation (5) from Figure 6. These rates are all above 12.5, indicating low mobility of heavy metals. This demonstrates that the combination of cement and ISS effectively immobilizes Cr, Pb, and Cu. This approach is highly significant for reducing the migration and diffusion rates of heavy metals in the environment, thereby mitigating soil and water pollution.

**Figure 8:**
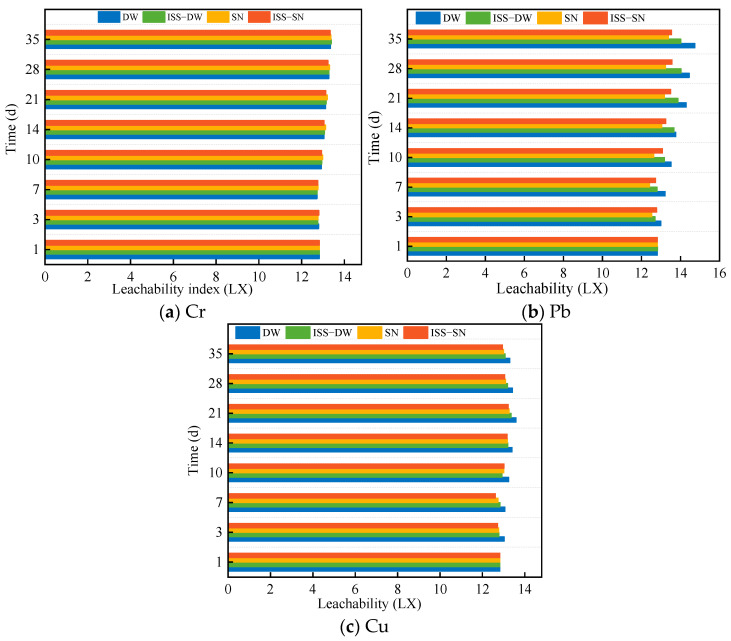
Leachability (LX) of (**a**) Cr, (**b**) Pb, and (**c**) Cu.

Additionally, to clarify the leaching risk associated with Cr, Pb, and Cu, and to determine the subsequent availability of red mud–graphite tailings, the LX value was calculated using Equation (6). The LX value is understood to inversely correlate with the leachability of heavy metals and the resultant risk of leaching, as demonstrated in Figure 8. An LX value exceeding 9 is indicative of a material suitable for recycling, for example, for use as green soil, fill beneath roadways, and construction materials. An LX value between 8 and 9 suggests that the material can be disposed of in landfills. In contrast, an LX value below 8 signifies the item’s unsuitability for landfill disposal [61]. In this investigation, the LX values for Cr, Pb, and Cu ranged from 12.71 to 13.38, 12.41 to 14.74, and 12.61 to 13.59, respectively. Although the research objects in comparative studies by Narasimman et al. [62] and Li et al. [63] were somewhat different from those analyzed here, they belong to the same category of industrial solid wastes. The LX values for Cr, Pb, and Cu were 9.01, 8.7, and 10.5, which indicates that Cr, Pb, and Cu were present in high-solidified red mud–graphite tailings. These ranges suggest that the materials in question have significant recyclability. Thus, it can be concluded that all the samples complied with landfill disposal criteria and were recyclable, enabling their classification as environmentally friendly and sustainable materials.

## 5. Conclusions

This study focused on the leaching behaviors and mechanisms of three metals (Cr, Pb, and Cu) in red mud–graphite tailings with cement analyzed using semi-dynamic leaching tests and modeling approaches. The main conclusions include the following:

(1) ISS significantly reduced the leaching of heavy metals. The specific mechanism through which ISS influences lead leaching in deionized water requires additional investigation.
(2) The experiments indicated that the leaching of Cr, Pb, and Cu follows the Elovich model and the double constant equation. The leaching behavior of Cr was also described by the Avrami equation. In line with the shrinkage core model, the leaching mechanism of heavy metal ions encompasses diffusion control, which is partly governed by a combination of internal diffusion and surface chemical reactions.
(3) Evaluation of the LX values of Cr, Pb, and Cu in the test samples indicated the potential reuse of red mud–graphite tailings. Thus, their application is environmentally sound and supports sustainable development efforts.

## Figures and Tables

**Table 1:** The elemental composition of red mud and graphite tailings.

Element	Mass Percentage (%)	
	Red Mud	Graphite Tailings
Ca	1.12	0.02
Na	8.64	0.74
Al	22.91	11.87
Si	11.62	39.37
Fe	36.6	11.55
Ti	6.45	0.74
Mg	0.08	4.52
Cr	1.12	0.02
Cu	0.01	0.02
Sr	0.004	0.02
Zr	0.19	0.03
Ti	6.45	0.73
Pb	0.01	0.006

**Table 2:** Results of USCM fitting for heavy metal leaching.

Element	Type	Mechanism	Correlation	Parameter
Cr	DW	internal diffusion	0.98	k = 0.00886
	ISS-DW	mixture of interfacial chemistry and diffusion	0.98	k = 0.00255
	SN	internal diffusion	0.97	k = 0.00881
	ISS-SN	internal diffusion	0.98	k = 0.00886
Pb	DW	mixture of interfacial chemistry and diffusion	0.85	k = 1.69484 × 10^4^
	ISS-DW		0.88	k = 2.18592 × 10^4^
	SN		0.90	k = 2.64448 × 10^4^
	ISS-SN		0.92	k = 2.47926 × 10^4^
Cu	DW	mixture of interfacial chemistry and diffusion	0.84	k = 6.84286 × 10^7^
	ISS-DW		0.85	k = 3.18006 × 10^7^
	SN		0.85	k = 7.49356 × 10^7^
	ISS-SN		0.80	k = 8.8319 × 10^7^

## Data Availability

The data are contained within the article. Additional data are available upon request from the corresponding authors.
